# Challenges Related to Informal Care Partners’ Involvement in End-of-Life-Care in Assisted Living

**DOI:** 10.1093/geroni/igaa057.054

**Published:** 2020-12-16

**Authors:** Molly Perkins, Ann Vandenberg, Candace Kemp, Mary Ball, Joanna Jungerman, Elise Abken, Alexis Bender

**Affiliations:** 1 Emory University, Atlanta, Georgia, United States; 2 Emory University School of Medicine, Atlanta, Georgia, United States; 3 Georgia State University, Atlanta, Georgia, United States

## Abstract

Limited empirical evidence suggests that caregiver burden is greater for informal care partners (family and friends) in assisted living (AL) compared with other long-term care settings, particularly within context of end of life. Using qualitative data from a larger 5-year, 7-site study of end-of-life care in AL funded by the National Institute on Aging (R01AG047408), we investigate informal care partners’ involvement in end-of-life care and identify challenges related to informal caregiving that might contribute to care burden. Grounded theory analysis of ethnographic data and in-depth interviews (average interview length = 97 minutes) with 59 racially and ethnically diverse informal care partners (mean age = 60) shows that informal care partner involvement in end-of-life care varies across participants and over time and is shaped by multiple intersecting social and structural determinants. At individual levels, these include many personal, situational, and relational factors. Personal factors include but are not limited to care partners’ own physical and mental health and material resources (e.g., ability to pay for supplementary care). Situational and relational factors include care partners’ awareness (or lack thereof) of residents’ impending death and the quality of the caregiving relationship. AL and wider community-level factors include understaffing, staff turnover, inadequate hospice support, and lack of access to these services. We find that informal care partners navigate these caregiving challenges through a basic social process we conceptualize as “negotiating risks.” Strategies for easing caregiver burden and improving informal care partner and resident quality of life at end of life are implicated.

